# Supplementary material for: Empirical methods for controlling false positives and estimating confidence in ChIP-Seq peaks
Source: BMC Bioinformatics. 2008 Dec 5;9:523. doi: 10.1186/1471-2105-9-523 (PMC2628906; doi:10.1186/1471-2105-9-523)
Supplement: Additional File 1 — A variety of html documents from the USeq web site detailing the available applications, their best usage, output file type descriptions, command line menus, etc. [file 1471-2105-9-523-S1.zip › USeqUserGuides/JavaDoc.zip!/JavaDoc/trans/main/WilcoxonRankSumTest.html]

WilcoxonRankSumTest


|  |  |  |
| --- | --- | --- |
| **Overview** \| **Package** \| **Class** \| **Use** \| **Tree** \| **Deprecated** \| **Index** \| **Help** | |  |
| **PREV CLASS**   **NEXT CLASS** | **FRAMES**    **NO FRAMES**     **All Classes** |  |
| SUMMARY: NESTED \| FIELD \| CONSTR \| METHOD | DETAIL: FIELD \| CONSTR \| METHOD |  |


---


## trans.main Class WilcoxonRankSumTest

```
java.lang.Object
  trans.main.WilcoxonRankSumTest
```

---

``` public class WilcoxonRankSumTest extends java.lang.Object ```

Implementation of a Wilcoxon Rank Sum Test.
Note, if the number of samples for either the treatment or control group is <10,
no test statistics are calculated and the pValue is set to 1, logTPvalue is set to 0,
and z to -10.

---

**Constructor Summary**

`WilcoxonRankSumTest()`


| **Method Summary** | |
| --- | --- |
| `double` | `calculateZ()`             Only valid when # treatment samples and # control samples >9 each. |
| `double` | `getLogTPvalue()` |
| `int` | `getNumberTreatmentsControls()` |
| `static void` | `main(java.lang.String[] args)` |
| `void` | `makeSamples(float[][] treatment, float[][] control, int startIndex, int stopIndex)`             Builds samples from part of a float[replica][intensities] for treat and control. |
| `void` | `makeSamples(float[] treatment, float[] control)` |
| `void` | `rankSamples()`             Ranks a sorted array of WilcoxonSamples based on value. |
| `void` | `setTwoTailed(boolean twoTailed)` |
| `void` | `sumRanks()` |
| `double` | `test(float[][] treatment, float[][] control, int startIndex, int stopIndex)`             Returns a -10LogBase10(pValue) if n1 and n2 >9 |
| `double` | `test(float[] treatment, float[] control)`             Returns a -10LogBase10(pValue) if n1 and n2 >9. |

**Methods inherited from class java.lang.Object**

`equals, getClass, hashCode, notify, notifyAll, toString, wait, wait, wait`

**Constructor Detail**

### WilcoxonRankSumTest

```
public WilcoxonRankSumTest()
```


**Method Detail**

### test

```
public double test(float[] treatment,
                   float[] control)
```

:   Returns a -10LogBase10(pValue) if n1 and n2 >9.
    if twoTailed is true and z is neg, will return a - logTPValue.

---


### test

```
public double test(float[][] treatment,
                   float[][] control,
                   int startIndex,
                   int stopIndex)
```

:   Returns a -10LogBase10(pValue) if n1 and n2 >9

---


### calculateZ

```
public double calculateZ()
```

:   Only valid when # treatment samples and # control samples >9 each.

---


### sumRanks

```
public void sumRanks()
```

---


### rankSamples

```
public void rankSamples()
```

:   Ranks a sorted array of WilcoxonSamples based on value.
    If no ties are found then this is simply their array index number+1.
    (ie 1,2,3,4...)
    If ties are encountered, ties are assigned the average of their index
    positions+1. (ie if index+1's: 2,3,4 have the same absolute difference, all are assigned a
    rank of 3).

---


### makeSamples

```
public void makeSamples(float[] treatment,
                        float[] control)
```

---


### makeSamples

```
public void makeSamples(float[][] treatment,
                        float[][] control,
                        int startIndex,
                        int stopIndex)
```

:   Builds samples from part of a float[replica][intensities] for treat and control.

    :   **Parameters:**: `float[replicas][intensities]` -

---


### main

```
public static void main(java.lang.String[] args)
```

---


### getLogTPvalue

```
public double getLogTPvalue()
```

---


### getNumberTreatmentsControls

```
public int getNumberTreatmentsControls()
```

---


### setTwoTailed

```
public void setTwoTailed(boolean twoTailed)
```


---


|  |  |  |
| --- | --- | --- |
| **Overview** \| **Package** \| **Class** \| **Use** \| **Tree** \| **Deprecated** \| **Index** \| **Help** | |  |
| **PREV CLASS**   **NEXT CLASS** | **FRAMES**    **NO FRAMES**     **All Classes** |  |
| SUMMARY: NESTED \| FIELD \| CONSTR \| METHOD | DETAIL: FIELD \| CONSTR \| METHOD |  |


---
